# Influence of Living Alone or with a Spouse Only on the Short-Term Prognosis in Patients after an Acute Ischemic Stroke

**DOI:** 10.3390/ijerph17218223

**Published:** 2020-11-06

**Authors:** Yohei Ishikawa, Toru Hifumi, Mitsuyoshi Urashima

**Affiliations:** 1 Department of Emergency and Critical Care Medicine, St. Luke’s International Hospital, Tokyo 104-8560, Japan; hitoru@luke.ac.jp; 2 Division of Molecular Epidemiology, The Jikei University School of Medicine, Tokyo 105-8461, Japan; urashima@jikei.ac.jp

**Keywords:** family, living arrangements, social isolation, socioeconomic status, stroke

## Abstract

Background—This study aimed to explore whether living alone or with a spouse only affects the short-term prognosis of acute ischemic stroke patients. Methods—We conducted a retrospective cohort study of patients with a diagnosis of acute ischemic stroke from April 2014 to February 2019 in Japan. The primary outcome was defined as worsening by at least one grade on the modified Rankin Scale (mRS). The secondary outcome was set as the degree of worsening on the mRS. The outcomes were compared between three groups of patients: (1) those living alone (ALONE), (2) those living with their spouse only (SPOUSE), and (3) OTHERs. Results—In total, 365 patients were included in this study: 111 (30%) ALONE, 133 (36%) SPOUSE, and 121 (33%) OTHERs. Cardiogenic embolisms were observed more frequently in ALONE than in OTHERs. The primary outcome occurred in 88 (79.3%) patients in ALONE and in 96 (72.2%) patients in SPOUSE, both of which were higher than the 72 (59.5%) in OTHERs. After adjusting with 19 variables, the risk of worsening was higher in ALONE (odds ratio (OR): 2.90, 95% confidence interval (CI): 1.50–5.58) and SPOUSE (OR: 1.83, 95% CI: 1.00–3.33) compared with OTHERs. Conclusions—In patients with acute ischemic stroke, not only living alone but also living with a spouse only may be associated with a worse short-term prognosis, independent of other cardiovascular risks.

## 1. Introduction

Low socioeconomic status is considered to increase stroke mortality, as well as its incidence [1]. Recent evidence on socioeconomic status and stroke shows that both short- and long-term prognoses after a stroke disproportionately affect not only low- and middle-income countries but also socioeconomically deprived populations within high-income countries [2]. Indeed, stroke remains one of the leading causes of disability and mortality in Japan, which is a high-income country that has a universal healthcare system. In fact, the 2-year cumulative mortality after a patient’s first stroke in Japan is still high (>30%) [3], although most patients at high risk for stroke, e.g., those with hypertension, diabetes, hyperlipidemia, or atrial fibrillation [4], are considered to be well cared for under the community health system. Stroke accounted for the highest percentage (51.1%) of the main causes of needing long-term care among those aged 40–64 years old in Japan [5]. Japan has become a super-aging society with an increasing number of nuclear families, where the number of older people living alone or with a spouse only is increasing. The percentage of households with someone aged 65 or older has been increasing every year, accounting for 48.9% of all households as of 2018 [6]. In addition, there has been a major change in the household structure. In 1980, three-generation households made up the largest proportion of the household structure, accounting for half of all households, but in 2018, the largest proportion of households were headed by a husband and wife alone, accounting for about 30% of all households, and when combined with single households, the figure was about 60% [6]. There has been a significant increase in the number of both men and women aged 65 and older living alone, from 4.3% of men and 11.2% of women in 1980 to 13.3% of men and 21.1% of women in 2015 [6]. However, to our knowledge, no study has assessed the influence of cohabitation status on stroke prognosis. Therefore, we conducted a retrospective cohort study of patients with acute ischemic stroke admitted at a single institute in Tokyo, Japan, to examine whether cohabiting family members could influence the short-term prognosis, as estimated by a worsening on the modified Rankin Scale (mRS) before stroke onset and at discharge.

## 2. Materials and Methods

### 2.1. Design and Overview

The data that support the findings of this study are available from the corresponding author on reasonable request. This retrospective cohort study was based on data obtained from the medical records of inpatients treated at St. Luke’s International Hospital in central Tokyo. Patients who presented by ambulance and were admitted to the hospital with a suspected acute ischemic stroke between 1 April 2014 and 28 February 2019 were included. All patients with clinical symptoms were diagnosed as having had an ischemic stroke by a neurologist or neurosurgeon, and confirmation was made based on findings from magnetic resonance imaging with fluid-attenuated inversion recovery and diffusion-weighted imaging. Among these patients, cases with other diseases, namely, a transient ischemic attack, epilepsy, and peripheral vertigo were excluded. The requirement for informed consent was waived because the data were anonymized and retrospective. All data were subject to strict privacy policies and all patients or their family members were given the chance to opt out. This study was approved by the Institutional Review Board at St. Luke’s International Hospital.

### 2.2. Cohabiting Family Members

Cohabiting family members, including a spouse (wife/husband) or partner, daughter/son (including daughter/son-in-law), and another family member(s) (e.g., sister/brother) who were living with the patients were prospectively and routinely interviewed at admission. Patients who had been living in a nursing home were counted as living alone, unless they were living with a family member at the nursing home. In terms of mutually exclusive factors, we classified patients into the following three groups: (1) patients living alone (ALONE); (2) patients living with their spouse only (SPOUSE); (3) patients living with at least one other family member, e.g., spouse and child (OTHERs).

### 2.3. Covariate Assessment

The covariates originally selected were age, sex, date of admission, date of discharge, discharge condition (alive or dead), current smoking status, comorbid conditions (e.g., diabetes mellitus, hypertension, dyslipidemia, and atrial fibrillation), drug usage (e.g., anticoagulants and antiplatelets), mRS score before onset as judged by a family member(s) and at discharge as judged by a physical therapist or doctor, National Institute of Health Stroke Scale (NIHSS) score at admission, cardiogenic embolism, wake-up stroke, treatment (e.g., administration of tissue plasminogen activator (tPA) and execution of mechanical thrombectomy), time from onset to the ambulance call, time from ambulance call to arriving at the hospital, and laboratory data at admission (e.g., white blood cells (WBC), hemoglobin (Hb), C-reactive protein (CRP), total protein (TP), albumin (Alb), blood urea nitrogen (BUN), and creatinine (Cr)).

### 2.4. Outcomes

The primary outcome was set as a worsening by at least one grade on the mRS at discharge compared with before the stroke onset. The mRS measures functional independence on a seven-grade scale, from 0 (no symptoms) to 5 (severe disability; bedridden, incontinent, and requiring constant nursing care and attention) and 6 (dead) [7]. Patients whose mRS score at discharge was worse compared with before the stroke onset were classified as “1” in accordance with the binary outcome. The secondary outcome was set as the degree of worsening: (mRS score at discharge)—(mRS score before the stroke onset).

### 2.5. Statistical Analysis

A logistic regression model and an ordered logistic regression model were applied to compute the odds ratios (ORs) and 95% confidence intervals (95% CIs) for the primary binary outcome and secondary ordered outcomes, respectively, with or without an adjustment using the following 19 variables: (1–4) age quartiles; (5) sex; (6) current smoking status; comorbidity of (7) hypertension, (8) dyslipidemia, (9) diabetes, and (10) atrial fibrillation; (11) usage of anticoagulants; (12) antiplatelets; (13) NIHSS at admission; (14) cardiogenic embolism; (15) wake-up stroke; (16) administration of tPA; (17) execution of mechanical thrombectomy; (18) time from onset to the ambulance call; (19) time from the ambulance call to arriving at the hospital. Age and creatinine and albumin levels were not normally distributed; therefore, the Kruskal–Wallis test was applied. Sex, hypertension, diabetes, atrial fibrillation, and current smoking were binary data; therefore, the chi-squared test was applied. When the 95% CI did not include 1, the OR or risk ratio (RR) was considered statistically significant. Time and laboratory data were compared between the three groups using the Kruskal–Wallis test and a test for trends [8]. Associations between laboratory data and the NIHSS scores at admission were analyzed using a linear regression model. In this analysis, results with two-sided *p*-values < 0.05 were considered statistically significant. All analyses were conducted using STATA 14.0 software (STATA Corp., College Station, TX, USA).

## 3. Results

### 3.1. Patients’ Characteristics

**Figure 1:**
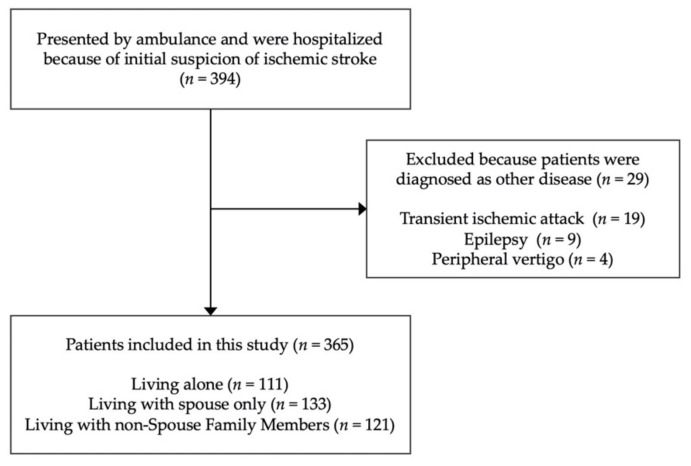
Flow diagram of the patient exclusion process.

From April 2014 to February 2019, 394 patients were presented by ambulance and were hospitalized because of initial suspicion of an ischemic stroke. However, 29 patients were excluded because their diagnosis turned out to be a different disease: 19 cases of transient ischemic attack, six cases of epilepsy, and four cases of peripheral vertigo. Finally, a total of 365 patients (238 (65%) male, median age: 74 years, interquartile range (IQR): 63–82 years) were enrolled in this study (Figure 1). A comparison of the patients’ characteristics between the three groups (ALONE, SPOUSE, and OTHERs) is shown in Table 1. The majority of patients in OTHERs group were living with either one or two family member(s), while a minority were living with three family members. The patients in the OTHERs group were living with a son (43%) and/or daughter (49%), with a spouse (40%) or without a spouse (60%). The proportion of males was highest in the SPOUSE group. The patients in the OTHERs group had worse mRS scores before the stroke onset. Cardiogenic embolism was observed more frequently in the ALONE group (34 (30.6%)) than in the OTHERs group (22 (18.2%); OR: 1.99, 95% CI: 1.03–3.86). No other differences were seen between the groups in the other factors, including the proportion of wake-up strokes, NIHSS score at admission, tPA use, and the implementation of a mechanical thrombectomy.

### 3.2. Differences in the Time from the Stroke Onset to Arriving at the Hospital

**Figure 2:**
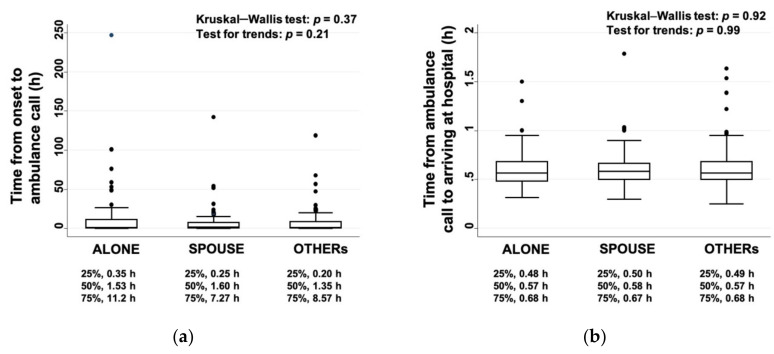
Cohabiting family members and the time from stroke onset to hospital admission: (**a**) time from the onset to the ambulance call and (**b**) time from the ambulance call to arriving at the hospital.

We hypothesized that cohabiting family members would notice signs of a stroke and call an ambulance, and thus, that patients living alone may be associated with a longer time from onset to call. However, no differences were found between the three groups in the time from the onset to the ambulance call or in the time from the ambulance call to arriving at the hospital (Figure 2). Overall, 60% of the patients in the ALONE group, 65% of those in the SPOUSE group, and 60% of those in the OTHERs group arrived at the hospital within 4.5 h from stroke onset.

### 3.3. Differences in the Laboratory Data at Admission

**Figure 3:**
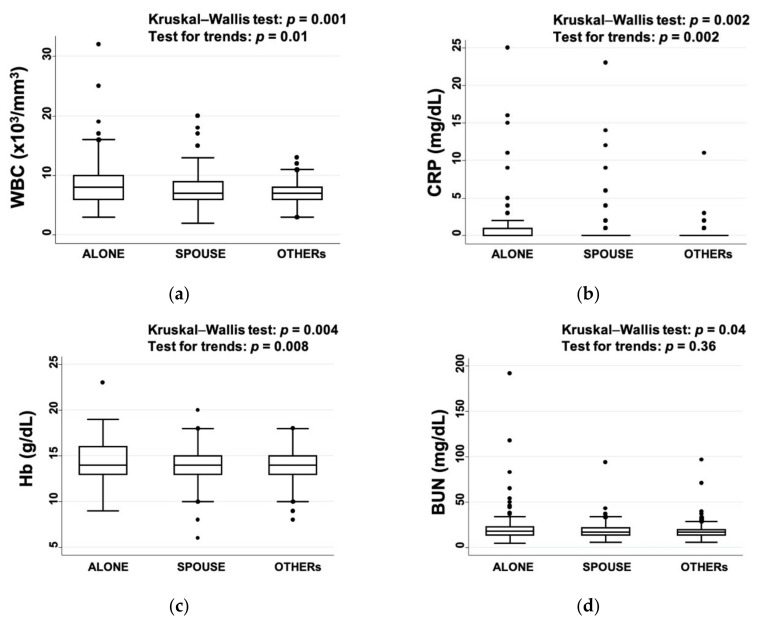
Laboratory data at admission: (**a**) white blood cells (WBC), (**b**) C-reactive protein (CRP), (**c**) hemoglobin (Hb), and (**d**) blood urea nitrogen (BUN).

The WBC, CRP, and Hb values measured at admission were the highest in the ALONE group, followed by the SPOUSE and OTHERs groups (Figure 3). The NIHSS scores at admission were significantly positively associated with WBC (*p* < 0.001) and CRP (*p* = 0.01), but not with any other factors. On the other hand, the TP, Alb, BUN, and Cr values were not significantly different between the three groups based on the Kruskal–Wallis test for trends.

### 3.4. Risk of Worsening by at Least One Grade on the mRS

**Figure 4:**
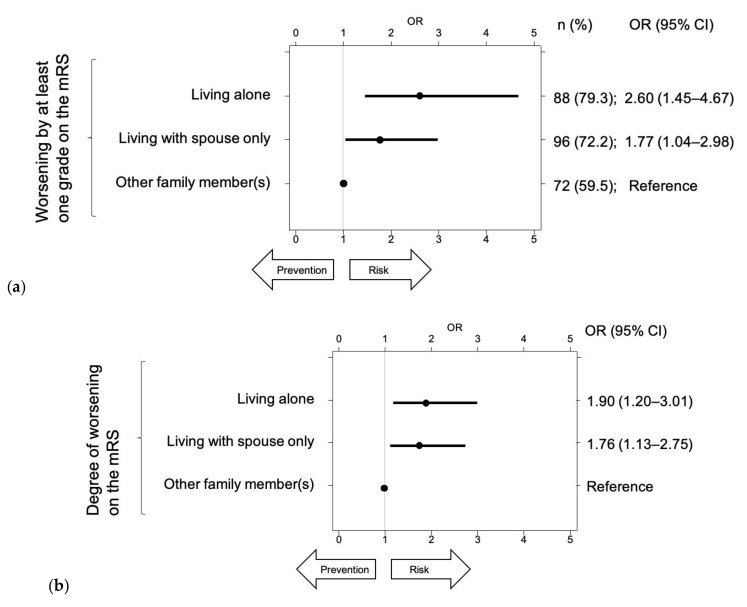
Odds ratios (ORs) for worsening mRS scores after a stroke: (**a**) worsening by at least one grade on the mRS after the stroke onset and (**b**) the degree of worsening on the mRS.

In total, 17 (4.7%) patients died after admission. The risk of death was higher in the ALONE group (9 (8.1%)) than in the OTHERs group (2 (1.7%); RR: 0.20, 95% CI: 0.05–0.92), but not significantly different compared with the SPOUSE group (6 (4.5%)). In total, 109 (29.9%) patients were discharged from the hospital without a reduction in the mRS score; in other words, the mRS scores were worse by at least one grade at discharge in the other 256 (70.1%) patients. The primary outcome, i.e., worsening by at least one grade on the mRS, was seen in 88 (79.3%) patients in the ALONE group and 96 (72.2%) in the SPOUSE group, both of which were significantly higher compared with the 72 (59.5%) in the OTHERs group (Figure 4a). Regarding the secondary outcome, i.e., the degree of worsening on the mRS, patients in the ALONE (OR: 1.90, 95% CI: 1.20–3.01) and SPOUSE groups (OR: 1.76, 95% CI: 1.13–2.75) showed significantly greater worsening than those in the OTHERs group (Figure 4b).

**Figure 5:**
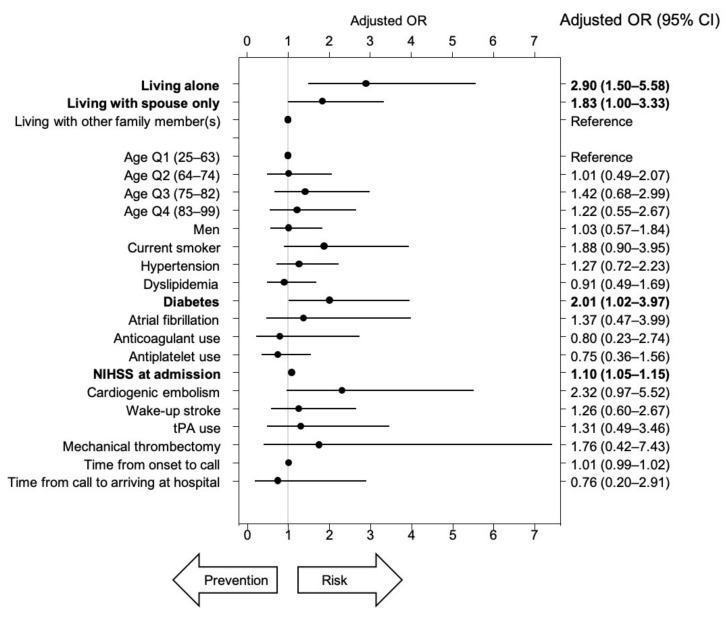
Adjusted odds ratios for worsening by at least one grade on the mRS after the stroke onset. tPA: tissue plasminogen activator. Bold type shows significant differences in the 95% CI.

**Figure 6:**
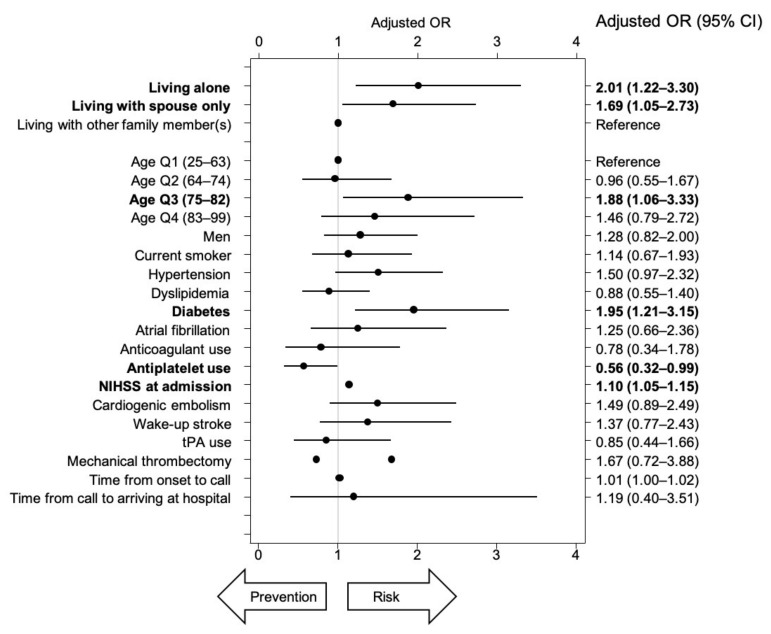
Adjusted odds ratios for changes in the mRS scores after the stroke onset ((mRS score at discharge)—(mRS score before stroke onset)). Bold type shows significant differences in the 95% CI.

Even after adjusting with 19 variables, the risk of worsening by at least one grade on the mRS was significantly higher in the ALONE (OR: 2.90, 95% CI: 1.50–5.58) and SPOUSE groups (OR: 1.83, 95% CI: 1.00–3.33) than in the OTHERs group (Figure 5). In a multi-logistic regression model, having diabetes and the NIHSS score at admission were also identified as significant risk factors. Even after adjusting with the same 19 variables, the degree of worsening on the mRS was also significantly more prevalent in the ALONE (OR: 2.01, 95% CI: 1.22–3.30) and SPOUSE groups (OR: 1.69, 95% CI: 1.05–2.73) than in the OTHERs group (Figure 6). In the multi-logistic regression model, age quartile 3, having diabetes, and the NIHSS score at admission were also identified as significant risk factors, whereas antiplatelet use was found to be a protective factor.

## 4. Discussion

To our knowledge, this is the first study in the world that was designed to examine the influence of cohabitation status, i.e., to conduct a comparison between patients living alone, with a spouse only, or with others, in terms of the short-term prognosis of an acute ischemic stroke. The primary outcome, i.e., worsening by at least one grade on the mRS at discharge compared with before the stroke onset, was observed in approximately 80% of patients in the ALONE group and 70% of those in the SPOUSE group, both of which were higher than that in the OTHERs group (60%). The secondary outcome, i.e., degree of worsening on the mRS, was also higher in the ALONE and SPOUSE groups than in the OTHERs group. Even after multivariate analyses, being in the ALONE or SPOUSE groups remained risk factors for both outcomes. Previous studies have reported that living alone is associated with increased long-term mortality after an ischemic stroke [9,10]. Another study reported negative results between living alone and mortality or readmission [11], but also that patients living alone were less likely to return home. In this study, we showed that not only living alone but also living with a spouse only were risk factors for short-term functional outcomes after acute ischemic stroke, although no significant difference in mortality was observed.

In this study, the time from the stroke onset to the ambulance call did not differ between the three groups. Patients living alone were found to be less likely to arrive at the hospital within 2.5 h, to receive thrombolysis, or to be discharged home [11], but we could not confirm this result. This study was carried out at a single institution located in a densely populated area, which may offset the time difference. Factors other than cohabitation may also be relevant, with one study showing that small and close-knit social networks had a delayed hospital arrival because of restricted information flow that reinforced the norm of watch-and-wait [12]. The frequencies of administration of tPA and execution of mechanical thrombectomy did not differ between the three groups, which supports the hypothesis that the influence of cohabitation status on worsening mRS scores may be independent of both the time from the stroke onset to arriving at the hospital and the initial treatments performed. As part of the long-term care insurance system in Japan, depending on the level of needed care, the staff of public community centers will visit the homes of elderly people living alone [13]. This system might have contributed to the lack of differences between the three groups in the time from the stroke onset to hospital arrival or the rate of administering tPA and/or a mechanical thrombectomy.

The WBC, CRP, and Hb values were the highest in the ALONE group, followed by the SPOUSE and OTHERs groups. A review of several previous studies indicated that high CRP values resulted in unfavorable long-term functional outcomes [14]. Indeed, the WBC and CRP values were associated with the NIHSS score at admission. A previous study reported that people who perform kind acts for others show favorable changes in genes that control inflammation [15]. Communication with a variety of people in the OTHERs group may have contributed to favorable changes in inflammation and fibrinolytic activity (plasmin–antiplasmin complex), as well as in subclinical atherosclerosis, which reduces the severity of symptoms after a stroke. In terms of Hb, both higher and lower Hb levels have been reported to be associated with higher mortality in patients after an acute ischemic stroke [16]. Therefore, the higher Hb levels observed in the patients in the ALONE group may have been indirectly associated with worsening mRS scores.

This study had several limitations. First, this was a retrospective cohort study. Although almost no data were missing, variables associated with cardiovascular disease, such as body mass index, were limited. Moreover, patients were not always transported from home; they were sometimes transported from their workplace, although we were able to collect data on cohabitation status. Second, only the prognosis during admission was determined in this study; additional studies involving long-term follow-up are therefore warranted. Third, socioeconomic statuses, including education, occupation, and income, were not measured. Fourth, confounders such as excessive alcohol intake, smoking, obesity, and a sedentary lifestyle were not measured. Finally, due to the uniqueness of the Japanese healthcare system and the central Tokyo area, caution should be taken when generalizing the results of this study to other countries or even other areas of Japan.

## 5. Conclusions

The results of the present study suggest that not only living alone but also living with a spouse only, may be associated with a worse short-term prognosis, independent of other cardiovascular risks, in Japanese patients after acute ischemic stroke. However, to improve the generalizability of these findings, a multicenter, prospective, cohort study is needed.

## Figures and Tables

**Table 1:** Patients’ characteristics.

Variable	Total	Living Alone	Living with Spouse Only	Living with Non-Spouse Family Members	*p*-Value
	*n* = 365	*n* = 111	*n* = 133	*n* = 121	
Age, median (IQR) ^1^	74 (63–82)	73 (64–84)	74 (63–81)	76 (61–83)	0.786
Male, *n* (%) ^2^	238 (65.2)	69 (62.2)	104 (78.2)	65 (53.7)	<0.001
mRS score before onset, *n* (%) ^1^					0.044
0	307 (84.1)	92 (82.9)	120 (90.2)	95 (78.5)	
1	14 (3.8)	4 (3.6)	6 (4.5)	4 (3.3)	
2	13 (3.6)	7 (6.3)	3 (2.3)	3 (2.5)	
3	9 (2.5)	3 (2.7)	2 (1.5)	4 (3.3)	
4	13 (3.6)	3 (2.7)	1 (0.7)	9 (7.4)	
5	9 (2.5)	9 (2.5)	1 (0.7)	6 (5.0)	
Hypertension (%) ^2^	207 (56.7)	60 (54.1)	84 (63.2)	63 (52.1)	0.162
Diabetes (%) ^2^	81 (22.2)	24 (21.6)	30 (22.6)	27 (22.3)	0.984
Atrial fibrillation (%) ^2^	57 (15.6)	23 (20.7)	16 (12.0)	18 (14.9)	0.170
Current smoking (%) ^2^	62 (17.0)	24 (21.6)	22 (16.5)	16 (13.2)	0.232
Creatinine (μmol/L), median (IQR) ^1^	69 (58–87)	69 (58–90)	70 (59–88)	68 (55–83)	0.559
Albumin (g/dL), median (IQR) ^1^	4 (4–4)	4 (4–4)	4 (4–4)	4 (4–4)	0.214

^1^ Age and creatinine and albumin levels were not normally distributed; therefore, the Kruskal–Wallis test was applied; ^2^ Sex, hypertension, diabetes, atrial fibrillation, and current smoking were binary data; therefore, the chi-squared test was applied; mRS: modified Rankin Scale; NIHSS: National Institutes of Health Stroke Scale; IQR: interquartile range.
